# Hooked on zombie worms? Genetic blueprints of bristle formation in *Osedax japonicus* (Annelida)

**DOI:** 10.1186/s13227-024-00227-1

**Published:** 2024-06-04

**Authors:** Ekin Tilic, Norio Miyamoto, Maria Herranz, Katrine Worsaae

**Affiliations:** 1 Marine Biological Section, Department of Biology, University of Copenhagen, Copenhagen, Denmark; 2 Marine Zoology Department, Senckenberg Research Institute and Museum, Frankfurt, Germany; 3 X-STAR, Japan Agency for Marine-Earth Science and Technology (JAMSTEC), Yokosuka, Japan; 4 Area of Biodiversity and Conservation, Superior School of Experimental Science and Technology (ESCET), Rey Juan Carlos University, Móstoles, Madrid Spain

**Keywords:** Chaetae, Chaetogenesis, Biomineralization, Chitin, Chitin synthase

## Abstract

**Background:**

This study sheds light on the genetic blueprints of chaetogenesis (bristle formation), a complex biomineralization process essential not only for the diverse group of bristle worms (annelids) but also for other spiralians. We explore the complex genetic mechanisms behind chaetae formation in *Osedax japonicus*, the bone-devouring deep-sea worm known for its unique ecological niche and morphological adaptations.

**Results:**

We characterized the chaetal structure and musculature using electron microscopy and immunohistochemistry, and combined RNAseq of larval stages with in-situ hybridization chain reaction (HCR) to reveal gene expression patterns integral to chaetogenesis. Our findings pinpoint a distinct surge in gene expression during the larval stage of active chaetogenesis, identifying specific genes and cells involved.

**Conclusions:**

Our research underscores the value of studying on non-model, "aberrant" organisms like *Osedax*, whose unique, temporally restricted chaetogenesis provided insights into elevated gene expression across specific larval stages and led to the identification of genes critical for chaetae formation. The genes identified as directly involved in chaetogenesis lay the groundwork for future comparative studies across Annelida and Spiralia, potentially elucidating the homology of chaetae-like chitinous structures and their evolution.

**Supplementary Information:**

The online version contains supplementary material available at 10.1186/s13227-024-00227-1.

## Background

Chaetae, the characteristic bristles of segmented worms (Annelida), are a hallmark of this diverse metazoan taxon, encompassing approximately 22,000 species [1]. Serving as the "Swiss Army knife" of annelids, these extracellular, hard, chitinous structures equip the worms with a variety of 'tools' for different purposes. Used as ‘paddles’ for swimming or crawling, or as ‘hooks’ for navigating through burrows or tubes via peristalsis [2–4]. The remarkable morphological diversity of chaetae not only mirrors the variety in motility patterns and life strategies among annelids but also underscores their significance in the group’s diversification and evolutionary success. Beyond facilitating movement, chaetae aid in a range of functions critical to survival of these organisms, from soil grip by the nearly imperceptible tiny bristles of earthworms (Lumbricidae) to defense against predation by the spiny, calcareous chaetae of fire worms (Amphinomida) [5, 6] or the flat, petal-like chaetae that cover the dorsum of Chrysopetalidae forming a shiny golden armour [7]. Moreover, in some scale worms, like *Sthenelais berkeleyi,* chaetae can even play a key role in respiration [8]. The iridescence of the sea mouse's (*Aphrodita*) bristles have intrigued researchers in fields from structural coloration to bio-inspired design and exemplifies the functional and morphological versatility of chaetae, likened to glass fibers in their physical properties [9, 10].

All these strikingly diverse chaetal morphologies are formed primarily by one single cell, the chaetoblast. This remarkable cellular machinery is adept at generating highly complex biomineralized structures [11, 12] and orchestrating the highly dynamic process of chaetal formation (chaetogenesis). The process entails intricate modulation of the cytoskeleton and microvilli, offering general insights into the formation of chaetae-like chitinous hard structures in other metazoans, such as the setae of Brachiopoda [13], Kölliker’s organ in juvenile octopuses [14] or the gizzard teeth in Bryozoa [15]. Chaetogenesis has been morphologically characterized in a variety of annelid species [11, 12, 16–18] based on detailed histological and ultrastructural analyses. However, a thorough molecular characterization of chaetae formation is lacking. The Ph.D. Thesis of Zakrzewski [19] does identify several genes involved in chaetogenesis, yet these findings still remain unpublished. Other studies have also shown genes not specific to chaetae formation but with expression patterns overlapping partly with the chaetal sac regions, such as *engrailed*, *Hox*, *Notch*, *Delta* and *hes2* in *Capitella teleta* and *P. dumerilii* [20–22]. Leveraging extensive ultrastructural expertise and the latest molecular technological advancements, we have examined the larvae of the bone-devouring worm *Osedax* to uncover the genetic mechanisms governing chaetogenesis.

The annelid *Osedax* thrives in a unique ecological niche, with their unparalleled ability to obtain nutrients from sunken vertebrate bones on the ocean’s floor [23, 24]. This ability relies on a highly specialized symbiotic relationship with endosymbiotic, heterotrophic *Oceanospirillales* bacteria, hosted in the root tissue of the worms [24–27]. Furthermore, *Osedax* is also characterized by a high level of sexual dimorphism, where the free-moving dwarf males resemble microscopic larvae [28], while the macroscopic, sessile females maintain male harems in the mucus holster surrounding their trunk [28–30]. Similar to many annelids, *Osedax* also possesses chitinous chaetae, prominently observed in settling larvae and males [30]. The hooked chaetae aid to the movement of metamorphing male larvae toward the sessile female and attachment near the oviduct of her trunk [28–30]. The metamorphing female larvae may also use the chaetae as aid for attaching themselves to the vertebrate bone during metamorphosis [28]. However, unlike the majority of annelids, *Osedax* features a fixed set of 16 chaetae, which was recently shown to only develop once in an individual’s lifetime, in the late larval stage, and never replaced [28]. The restricted time frame of chaetal development in *Osedax* provides a significant advantange and opportunity for studying the genetic regulation of chaetogenesis. In this study, we build upon our recently described staging system of the *O. japonicus* life cycle [28] and we have sequenced the transcriptomes of early embryos, all five larval stages preceding metamorphosis, along with early and late males and females. Our aim is to explore the genetic mechanisms governing chaetae formation, capitalizing on this high-resolution transcriptomic dataset and the restricted timing of chaetogenesis in *Osedax japonicus*. Additionally, our study includes the morphological characterization of the chaetoblast and chaetal follicle cells, and employs in-situ hybridization chain reaction (HCR) [31, 32] to reveal the localized expression of genes in specific cells involved in chaetogenesis. This approach offers key insights into the genetic machinery driving chaetogenesis and demonstrates the utility of HCR as a valuable tool for studying chaetae formation.

## Results

### Structure of chaetae and chaetal follicles

**Fig. 1:**
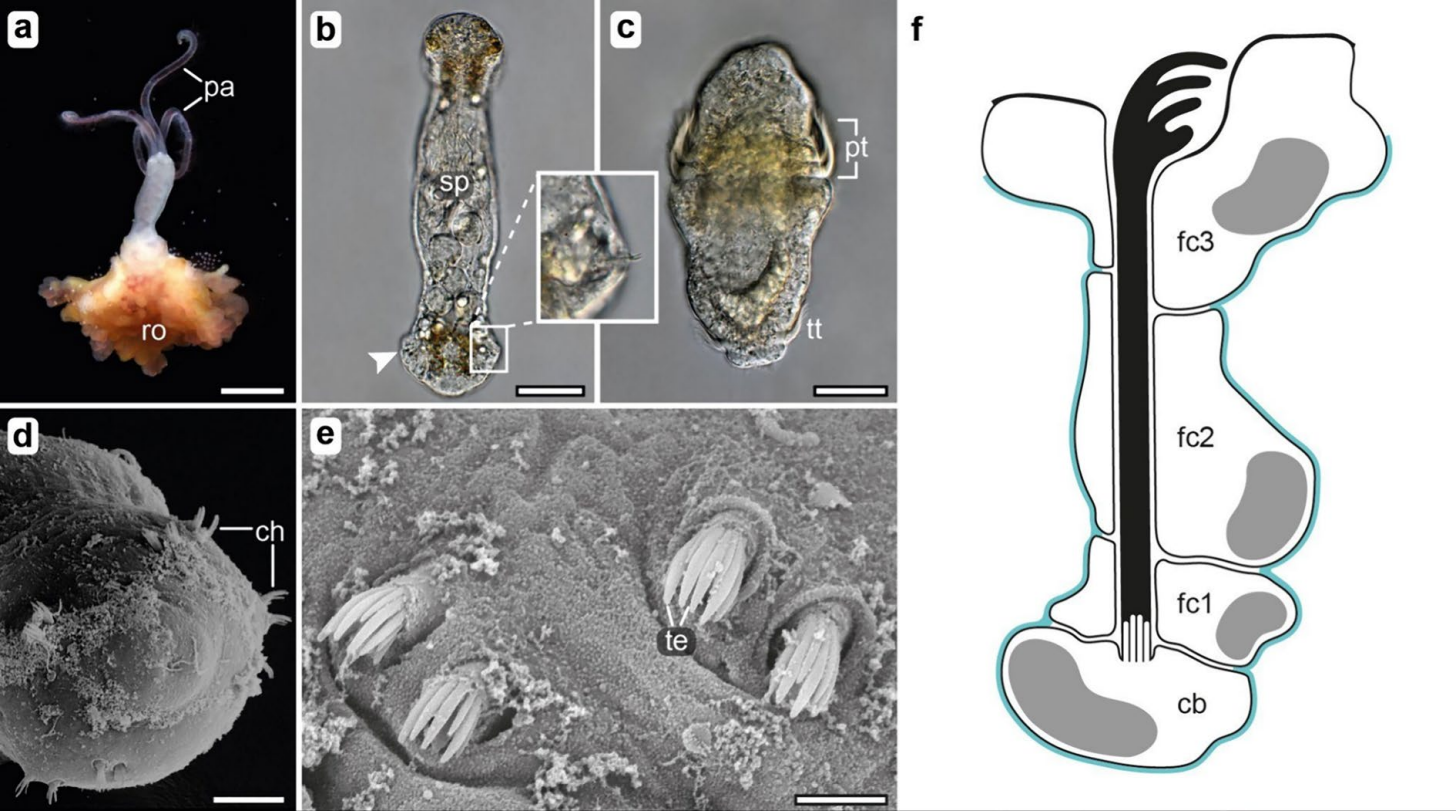
*Osedax japonicus* general and chaetal morphology. **a** Adult female *Osedax japonicus* with four elongate palps (*pa*) and root tissue (*ro*) dissected out of the bone. **b** Mature dwarf male with vital sperm (*sp*) within the body cavity. The position of posteriorly located chaetae are marked with *arrowheads*. **c** 5-day old competent trochophora larva, with 3 rows of ciliary bands in the prototroch (*pt*) and a single ciliary band in the posterior telotroch (*tt*). **d** Scanning electron microscopic (SEM) image of the posterior end of a late larva with hooked shaped chaetae (*ch*), arranged in pairs **e** SEM showing detail of the hooked chaetae, each with ca. 10 teeth (*te*). **f** Schematic drawing of a chaetal follicle with three follicle cells (*fc*) and the basal most chaetoblast (*cb*) with an apical array of dynamic microvilli. Abbreviations: *cb* chaetoblast, *ch* chaetae, *fc* follicle cell, *pa* palps, *pt* prototroch, *ro* root tissue, *sp* sperm, *te* teeth, *tt* telotroch. Scale bars: **a** 2 mm,** b** 50 µm, **c** 25 µm, **d** 10 µm, and **e** 2 µm

**Fig. 2:**
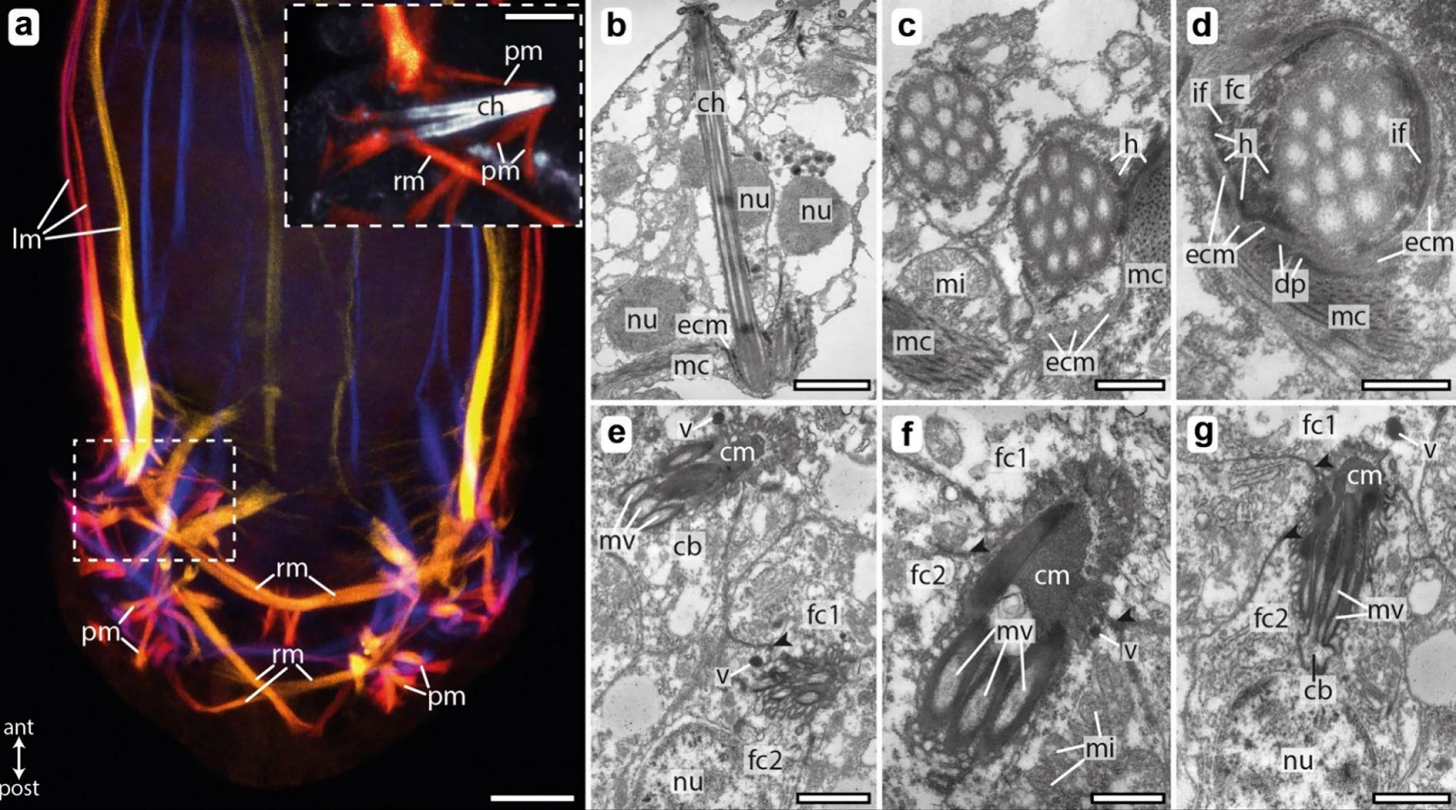
Chaetal musculature and ultrastructure of the chaetal follicle. **a** Confocal laser scanning microscopy (CLSM) of phalloidin stained musculature in a male *O. japonicus*, the z-projection is depth coded, and the inlet shows a higher magnification of a chaetal sac, with chaetal autofluorescence shown in gray. **b**–**g** Transmission electron microscopy (TEM) of fully developed and developing chaetae within chaetal follicles of 4-day old trochophore larvae. **b** Longitudinal section through a fully developed chaeta. **c** Transverse sections of two neighboring chaeta, within a single chaetal sac. **d** Detail of chaetal shaft near the base connected to the chaetal musculature via hemidesmosomes. **e** Two developing chaetae within a chaetal sac **f**, **g** Details of developing teeth. Abbreviations: *ant* anterior, *cb* chaetoblast, *ch* chaeta, *cm* amorphous chaetal material, *dp* dense plaques, *ecm* extracellular matrix, *fc* follicle cell, *h* hemidesmosomes, *if* intermediate filaments, *lm* longitudinal muscles, *mc* fiber muscle cell, *mi* mitochondria, *mv* microvilli, *nu* nucleus, *pm* protractor muscle, *post* posterior, *rm* retractor muscle, *v* electron-dense vesicle. Scale bars: **a** 10 µm, **b** 5 µm, **c** 500 nm, **d** 500 nm, **e** 1 µm, **f** 500 nm, and **g** 1 µm

The anatomy of an *Osedax japonicus* dwarf male (Fig. 1b) presents a striking deviation from the typical annelid body plan, bearing a closer resemblance to their larvae (Fig. 1c) rather than their female counterparts (Fig. 1a). These dwarf males, devoid of a mouth and gut, resemble the late-stage larvae arrested in development [28], utilizing their yolk reserves for sperm production, which nearly fills their entire body (Fig. 1b). However, akin to most annelids, these miniature male worms possess chaetae, which are maintained from the larval stage. *Osedax* chaetae are long-handled hooks emerging from the posterior two segments of the competent larvae, with a fixed set of 16 chaetae. With 8–10 teeth that curl apically, these hooks almost appear like tiny claws piercing through the body wall (Fig. 1e). Each segment is equipped with eight chaetae, arranged in two ventral (neuropodial) and two dorsal (notopodial) chaetal sacs (Fig. 1d, e). Chaetogenesis in *Osedax* is not a continuous process; instead, it occurs exclusively on the 4th day of larval development, irrespective of sex (as observed in Ref [28]). In young females, chaetae are also present but inconspicuous, found near the root–trunk transition, where they are unlikely to serve any apparent function. The role of chaetae in late larvae and males is clearly associated with locomotion. In contrast to the otherwise relatively simple fence-like body wall musculature observed in larvae [28] and males [30], a more intricate musculature comprising protractors and retractors governs the movement of chaetae (Fig. 2a). Each chaetal sac is supplied by approximately four short protractor muscles originating at its base and inserting at the body wall (Fig. 2a). The muscle fiber cells attach to the extracellular matrix (ecm), enveloping the epidermal chaetal sac, through hemidesmosomes located on the basal surface of the follicle cells. These structures link the intermediate filaments within the follicle cells to the ecm (Fig. 2c, d). Directly across from these hemidesmosomes, dense plaques of the muscle fiber cells also adhere to the ecm, creating a mechanical linkage that connects the muscle system directly to the chaeta. This connection is facilitated by the follicular intermediate filament system, which exhibits a densely packed arrangement of intermediate filament bundles (Fig. 2d). These filaments extend apically, anchoring to hemidesmosomes that, in turn, secure them to the chaeta, ensuring a robust connection between the muscle system and the chaetal follicles (Fig. 2d). Hence, follicle cells not only provide mechanical fixation of the chaetae but also attach the chaetae to the protractor muscles, ensuring their motility and protraction.

Transverse retractor muscles span the width of the body, connecting the chaetal sacs on each side. These muscles link the dorsal (notopodial) and ventral (neuropodial) chaetal sacs (Fig. 2a). Similarly, in the posterior segment, transverse muscle strands arise from the base of each chaetal sac, crossing over to insert at the body wall on the opposite side (Fig. 2a). The contraction of these muscle strands facilitates the retraction of the chaetae.

During chaetae formation in *O. japonicus*, the first structures to emerge are the curved teeth, each formed by a single apical microvillus extending from the chaetoblast (Fig. 2e, f). Below these curved teeth, a protrusion later forms, composed of a cluster of microvilli (Fig. 2e). As the formation of the hook's apical portion progresses, these microvilli align and elongate, forming a tightly organized bundle (Fig. 2c). As the chaeta extends outwards, the chaetoblast simultaneously moves downward, resulting in the gradual retraction of the microvilli bundle. This outward extension is driven by the polymerization of chitinous chaetal material along the microvillar template, ultimately creating the chaeta's lengthy shaft (Fig. 2b). The first and second follicle cells, positioned above the chaetoblast, encase the developing chaeta from either side and form a lumen around this (Fig. 2e–g). They are attached to the chaeta with static microvilli (Fig. 2f), which, unlike the dynamic apical microvilli of the chaetoblast, are not packed with actin filaments. Electron-dense vesicles that transport the chaetal material into the follicle’s lumen are observed near the adluminal surface of the follicle cells (Fig. 2e, f). Within the follicular lumen of the chaeta, a dense, amorphous chaetal material is secreted, forming a thick enamel layer that coats the external surface of the chaeta (Fig. 2f). The tips of the curved teeth are densely filled with chitinous material (Fig. 2f), contrasting with the shaft, which maintains hollow channels, remnants of the microvilli that were once inside the chaeta (Fig. 2b–d). This architectural difference likely imparts flexibility to the shaft while ensuring the rigidity and robustness of the apical teeth [16, 33].

### Differential gene expression across developmental stages and sexes

Our comparative transcriptomic analysis focused on three genes putatively associated with chaetogenesis—chitin synthase 1 (*CS1*), chitin synthase 2 (*CS2*), and neuronal cytoplasmic intermediate filament protein (*NF70*). Chitin synthases play a pivotal role in chaetae formation, due to their direct involvement in chitin production. In our transcriptome dataset for *Osedax japonicus*, we identified two chitin synthase genes: *OjapCS1* and *OjapCS2* (Suppl Fig. 1). Our phylogenetic analysis revealed that these genes are part of the spiralian chitin synthase groups A and B, respectively. *OjapCS1* clusters with *CateCS1* (from C*apitella teleta*), whereas *OjapCS2* groups within a cluster of annelid chitin synthases that includes *OwfuCS3* (*Owenia fusiformis*), *PlduCS3* (*Platynereis dumerilii*), *SaalCS3* (*Sabellaria alveolata*), and CateCS2 (*Capitella teleta*). The rest of the gene tree topology is consistent with findings presented in Ref. [34]. *NF70* was selected for its potential role in chaetogenesis, as suggested by single-cell RNA sequencing results from Ref. [35], which indicated its expression in chaetal sacs. Our phylogenetic analysis of *NF70* genes, extracted from public databases, revealed that *OjapNF70* clustered with *NF70* sequences from other annelids. This annelid group was identified as the sister group to the sole brachiopod *NF70* sequence available from *Lingula anatina*. Moreover, within the mollusks, two distinct groups of *NF70* were identified (Suppl Fig. 2).

**Fig. 3:**
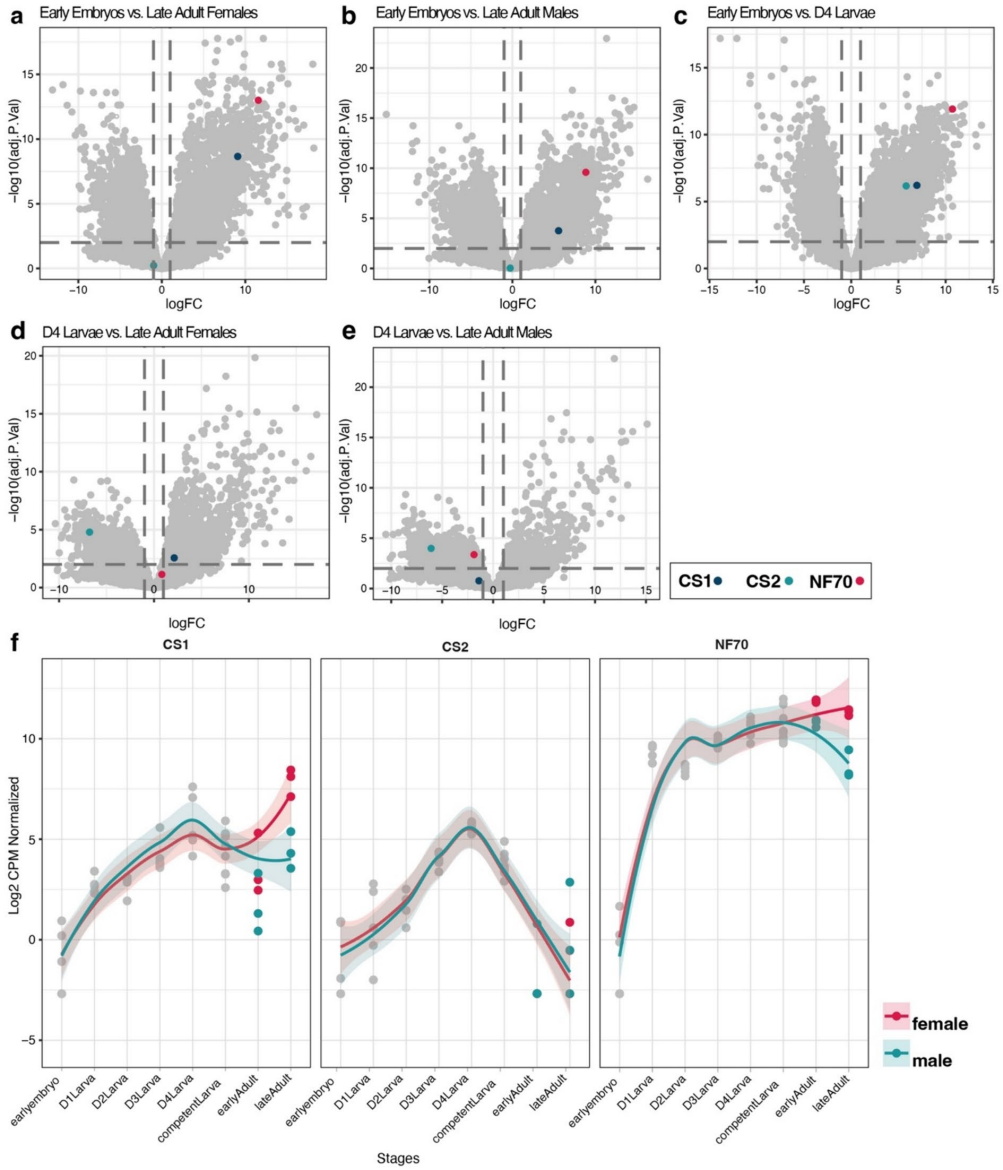
Gene expression patterns of chitin synthases (*CS1* and *CS2*) and of the neuronal cytoplasmic intermediate filament protein gene *NF70*. **a**–**e** Volcano plots showing log fold-change versus adjusted P values. **f** Normalized expression levels (log2 CPM normalized) of three genes (*CS1*, *CS2*, *NF70*) across different developmental stages and sexes. The trend in expression level is plotted separately for the two sexes

We uncovered distinct expression profiles for *CS1*, *CS2* and *NF70*, that vary across early embryos and subsequent developmental stages, and between sexes. Volcano plots (Fig. 3a–e, Suppl. Figure 3a, b) illustrate the magnitude and significance of differential gene expression across pairwise comparisons.

Our previous morphological study has identified day 4 of larval development as the critical juncture for chaetogenesis [28], and we therefore anticipated an upregulation of chaetogenesis-related genes in D4 larvae. Early embryos represent a baseline (or negative control) for gene expression, as they correspond to fertilized eggs in the initial cleavage stages, lacking chaetae or active chaetogenesis. Since adult males and females possess fully differentiated chaetae they were likewise not expected to exhibit an upregulation of chaetogenesis-related genes. The juxtaposition of early embryos with D4 larvae (Fig. 3c) did highlight a significant elevation in the expression of both *CS1*, *CS2* and *NF70*. Interestingly, the *CS1* and *NF70* were also upregulated in adult males and females compared to early embryos (Fig. 3a, b). *CS2* did not cross the threshold of significance in comparisons of early embryos with adult females and males (Fig. 3a, b).

The observed expression trends of *CS2* are further reinforced by its comparative analysis against other stages: D4 larvae versus late and early female adults (Fig. 3d, Suppl. Figure 3a), as well as versus late and early male adults (Fig. 3e, Suppl. Figure 3b). In these groups *CS2* consistently exhibits significant downregulation when measured against the expression levels at day 4 of larval development, indicating a pivotal role of *CS2* during this specific stage. *CS1* maintains expression levels below the threshold of significance in comparisons of D4 larvae with both adult males and juvenile females (Fig. 3e, f, Suppl. Figure 3a), with a modest increase noted in adult females (Fig. 3d, f). As for the *NF70* gene, a slight upregulation is observed in juvenile females in contrast to D4 larvae (Fig. 3g, Suppl. Figure 3a), while a marginal downregulation is apparent in adult males (Fig. 3e, f).

The developmental expression trajectories of *CS1*, *CS2*, and *NF70* become even more clear when they are profiled across all sampled stages and between sexes (Fig. 3f). *CS2* expression increases from the early embryo stage, culminating at the D4 larvae stage with a pronounced peak followed by a subsequent decline. *CS1*'s trajectory parallels *CS2*'s up to day 4, after which it demonstrates a more moderate decrease in males and a subtle rise in females. *NF70*'s expression profile is characterized by a sharper increase, nearly peaking by day one, and maintaining a relatively constant expression level thereafter. A slight upsurge in *NF70*'s expression levels is observed in adult females.

### Validation of localized gene expression with in-situ HCR

**Fig. 4:**
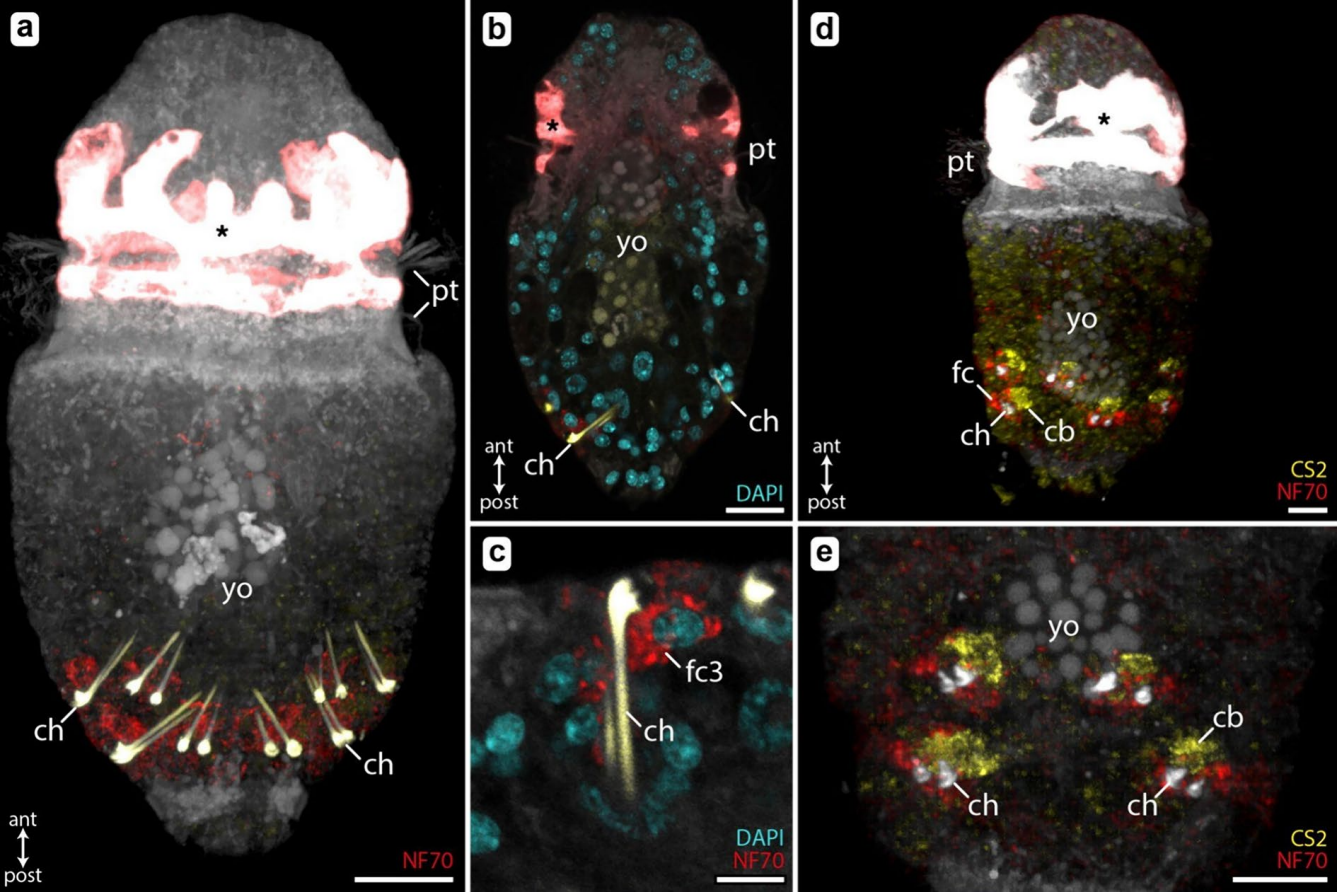
Localized expression patterns of chitin synthase (*CS2*; yellow) and neuronal cytoplasmic intermediate filament protein gene (*NF70*; red) validated by in-situ HCR. Chaetal autofluorescence is shown in white (**d**–**e**) and yellow (**a**–**c**), and nuclei (DAPI) in cyan. **a**–**c** Late day 4, competent larvae, with fully differentiated, long-shafted chaetae. **d**–**e** D4 larvae with active chaetogenesis, only the apical part of the chaetae is already formed. The autofluorescent, large pigmented cells of the prototroch are marked with an *. Abbreviations: *ant* anterior, *cb* chaetoblast, *ch* chaetae, *fc* follicle cell, *post* posterior, *pt* prototroch, *yo* yolk cells. Scale bars: **a** 15 µm, **b** 15 µm, **c** 5 µm, **d** 10 µm, **e** 10 µm

In-situ Hybridization Chain Reaction (HCR) experiments were employed to validate the spatial expression patterns of genes that exhibited elevated expression levels in our transcriptomic analysis. We did not detect any localized expression of *CS1*, as no positive signal was observed in any of the samples. In contrast, *CS2* and *NF70* presented a distinct expression within the chaetal follicles (Fig. 4), corroborating the gene expression profiles elaborated above.

*NF70* was consistently expressed in follicle cells during the process of chaetogenesis (Fig. 4d) and maintained expression in chaetal follicles post-formation in older competent larvae (Fig. 4a–c). The high, cell-level resolution of in-situ HCR allowed identifying the apical-most, third, follicle cells as the site of *NF70* expression in fully differentiated chaetae (Fig. 4c). It is not as clear whether follicle cells one and two are also expressing *NF70* during chaetae development (Fig. 4e).

Most strikingly, *CS2* demonstrated exclusive expression by the chaetoblast at the base of developing chaetae, with expression conspicuously limited to the period of active chaetogenesis (Fig. 4 d, e). This localized expression pattern of *CS2* is in exact agreement with the upregulated gene expression found in D4 larvae (Fig. 3).

## Discussion

### Structure, function and homology of *Osedax*’s hooks

*Osedax* belongs to Siboglinidae within the major annelid clade Sedentaria, comprising many burrowing and sessile annelids with hooked chaetae. The homology of hooked chaetae in sedentarian annelids is supported by the timing and spatial relationship of chaetal substructures [11, 16, 18, 36]. The sedentarian hooked chaetae are characterized by a distinct single large ‘tooth’ (rostrum), a cap of several smaller teeth (capitium), and a common shaft or manubrium [37]. Their development has been described to generally follow a "typical" pattern [18], which begins with formation of the rostrum, from a cluster of microvilli, followed by the capitium's teeth, each arising from individual wider microvilli. Subsequently, a multitude of small microvilli give rise to the subrostral process, with all microvilli aligning to form the shaft.

The scarcity of chaetae and the limited development within *Osedax* complicae the characterization of timing and patterns of chaetogenesis. The initial structures to emerge during *Osedax* chaetogenesis are curved teeth, each originating from a single microvillus, suggesting a homology with the capitium. An additional 'rostral' feature of the hook is observed beneath these teeth, composed of several microvilli. However, its later emergence during development questions its homology with the rostrum in hooked chaetae of other sedentarian annelids, and it may represent a subrostral process instead. If this is the case then the rostrum in *Osedax*’s chaetae would be reduced. The rostrum is also reduced within other sedentarian annelids such as Pectinariidae [38], a family-ranked taxon well nested within Terebellida, which otherwise primarily possess hooked chaetae with a rostrum [1, 16, 39]. Recent insights into annelid phylogeny coupled with new observations of remarkably different hook ultrastructure and formation [40], challenge the homology of all annelid hooks and substructures hereof. Nonetheless, targeted comparisons of chaetogenesis within closely related taxa and distinct monophyletic clades offer a framework for testing and potentially substantiating hypotheses of homology.

Chaetogenesis and chaetal ultrastructure have been investigated in other genera of Siboglinidae than *Osedax* [41]. In the opisthosomal chaetae of Vestimentifera, the posterior group of teeth has been homologized to the capitium, and the anterior group is suggested to represent a subrostral process [41]. However, vestimentiferan hooks also display a pronounced central protuberance which is suggested to likely correspond to the rostrum [41]. The sequence of chaetal development is crucial for homology assessments, and the lack of a distinct formative site and the serendipitous discovery of chaetal developmental stages via transmission electron microscopy (TEM) in Vestimentifera adds an additional layer of complexity to these characterizations and homology hypotheses. Further understanding of the genes involved in chaetae formation will provide an additional comparative framework and could help in resolving the homology of different chaetae in the future.

In the majority of sedentarian annelids, hooked chaetae serve a primary function in anchoring to the tube, facilitating the worm’s stability and movement within its habitat [3, 4, 42]. However, in *Osedax*, these structures clearly serve a different function. Here, the hooks are integral to locomotion, manipulated by a complex musculature network—arguably the most intricate aspect of the larval and male muscle system. This musculature enables precise movements of individual chaetae, which can be retracted and protracted in a controlled fashion. Male *Osedax* utilize their hooks to move through the mucus holster of the female and position themselves along her trunk and oviduct. In juvenile females undergoing metamorphosis, chaetae may aid in anchoring and burrowing into the bony substrate, indicating a potential secondary role in attachment during a critical life stage transition.

### Genes involved in chaetogenesis

The unique case of *Osedax*, characterized by its limited chaetogenesis that occurs only once within the lifetime of an individual, presents both advantages and challenges for the study of chaetal formation. Our comprehensive differential gene expression analysis enabled us to check for the up and downregulation of genes putatively involved in chaetogenesis across a developmental time series, as the time of chaetogenesis was known [28]. Given that chaetae are chitinous structures, the involvement of chitin synthase, the key enzyme in chitin secretion, is a logical assumption. From our transcriptomic dataset we identified two chitin synthase genes in *O. japonicus*, *CS1* and *CS2*.

*CS2* exhibited a notable increase in expression level on the fourth day of larval development, specifically during chaetal formation, with levels decreasing after this stage. This pattern suggests a direct involvement in chaetogenesis, a hypothesis we could test and corroborate in our in-situ Hybridization Chain Reaction (HCR) experiments. These experiments demonstrated that *CS2* is exclusively expressed in the chaetoblast and only during active chaetogenesis. *CS1*, on the other hand, showed an upregulation on day 4 and in female *Osedax*. The absence of a significant post-day-4 decline in expression implies that *CS1* may not be solely involved in chaetae formation. While in-situ HCR experiments using *CS1* probes yielded no positive signals near the chaetae or elsewhere, this does not definitively rule out its involvement in chaetogenesis. Chaetogenesis in most other annelids happens continuously within multiple segments, with different developmental stages of chaetae present simultaneously in each segment. In *Osedax*, chaetogenesis is a singular, time and spatially restricted event, which, on one hand offers unique insights into chaetal formation as demonstrated by our DGE results, but on the other hand makes it challenging to localize and determine the expression of genes involved in chaetogenesis with in-situ HCR experiments.

We did not conduct any in-situ HCR experiments in female *Osedax,* which leaves the observed slight elevation in *CS1* expression of females unexplained. Notably, in *Osedax* females a thin lining can be observed around the root tissue. The composition and origin of this lining, particularly whether it is chitinous and secreted by the worm itself, are yet to be determined. *CS1* might play a role in the secretion of this sheath. If this was the case, it would provide a potential explanation for the elevated *CS1* expression levels observed in adult female *Osedax*. Further investigation, including targeted experiments to ascertain the nature of this lining and the specific role of *CS1* in its formation, is needed to explore this possibility.

The third gene we investigated for its potential role in chaetogenesis was *NF70*, an intermediate filament protein known for its involvement in cytoskeletal processes and previously identified as expressed within the chaetal follicle [35]. Our DGE analysis revealed that *NF70*'s expression levels were relatively constant throughout development, with a notable increase from as early as day 1. This pattern suggests that *NF70*'s role extends beyond chaetogenesis, which is plausible given the widespread involvement of intermediate filaments and the cytoskeleton in various cellular and developmental processes. Intriguingly, our in-situ HCR experiments were able to shed light on the involvement of *NF70* in chaetogenesis. These experiments demonstrated that *NF70* is not only active during the formation of chaetae but also continues to be expressed after the completion of chaetogenesis. Specifically, in fully formed chaetae, *NF70* was observed to be expressed in the apical-most (third) follicle cell. This finding aligns with the previous ultrastructural studies, which have shown that follicle cells, often laden with intermediate filaments, play a crucial role in attaching and stabilizing the chaeta within the follicle [9, 11].

The results presented in this study represent a significant step forward in identifying genes involved in chaetogenesis. By utilizing differential gene expression analysis across a developmental time series, we have been able to pinpoint specific genes that play crucial roles in chaetae formation in *Osedax japonicus.* Moreover, the comprehensive RNA-seq dataset we have compiled is an invaluable resource for future studies exploring gene expression during the development and sexual maturation of *Osedax japonicus*. However, the need for other annelid model systems, particularly those with larger and more abundant chaetae and continuous chaetogenesis, is clear.

While a set of candidate genes involved in chaetogenesis has been previously proposed [19], they remain unpublished. The increasing availability of single-cell RNA-seq data in annelids [35] enhances the feasibility of screening for genes involved in chaetogenesis and other developmental processes. Our application of in-situ HCR in *Osedax* demonstrates that this technique is not only effective for confirming gene expression within the follicle but also provides cell-level resolution. This advancement is pivotal, allowing us to discern whether gene expression is restricted to specific cells, such as the chaetoblast or certain follicle cells. This has been a significant limitation in traditional in-situ hybridization techniques, where pinpointing exact cell-level gene expression has been challenging [19].

A better understanding of the genetic machinery regulating the intricate process of chaetogenesis that produces a diverse array of highly complex hard structures has far-reaching implications. Not only does it shed light on a sophisticated biomineralization process, potentially inspiring biomimetic designs, but it also aids in deciphering the homology of chitinous chaetae-like structures across other spiralian taxa.

## Methods

### Sample collection

The animals used in this study were obtained from the laboratory culture of *Osedax japonicus* Fujikura, Fujiwara & Kawato 2006 maintained at the Japan Agency for Marine-Earth Science and Technology (JAMSTEC). *Osedax japonicus* specimens were held in 100-L aquaria at a controlled temperature of 11 °C within the laboratory, where they grew on vertebrate bones. Embryos and males were collected from the mucus holster of mature females. Freshly laid, fertilized eggs were deposited in clusters by the females into the mucus. These eggs were carefully selected under a stereomicroscope, to ensure that no cleavage had occurred before isolating them into 200 ml dishes for subsequent sampling over a 1-week period. Larval settlement was induced by the introduction of fish scales and symbiotic bacteria into the dishes where the competent larvae (5–6 day old) were maintained. Detailed culturing conditions can be found in Refs. [27, 43], and larval staging follows what is outlined in Ref. [28].

### Transmission and scanning electron microscopy

The specimens intended for transmission electron microscopy (TEM) and scanning electron microscopy (SEM) were initially fixed using 2.5% glutaraldehyde buffered with 0.05 M phosphate buffer. Larvae were placed directly into the fixative and left to fix for 1 h at room temperature. Subsequently, they were rinsed in the same buffer 3 times for 1 h in total and stored in a buffer solution containing NaN_3_ until further processing. Prior to proceeding with dehydration using an ascending acetone series, the specimens were postfixed in 1% OsO_4_ buffered with PBS for 30 min (at 4 °C). Depending on the intended analysis, the specimens were either embedded in Araldite resin for sectioning or dried using hexamethyldisilazane (HMDS) for SEM. Silver-interference colored, ultra-thin sections (70–75 nm) were prepared from several 4-day-old and competent larvae using a diamond knife (Diatome) mounted on a LEICA U6 ultramicrotome and were subsequently placed on Formvar-covered, single-slot copper grids. Sections were stained with uranyl acetate and lead citrate in an automated TEM stainer (QG-3100, Boeckeler Instruments) and then examined using a ZEISS EM 10 electron microscope with phosphor imaging plates (Ditabis). Larvae that were dried using HMDS were sputter coated with a layer of gold–palladium (Au–Pd) and imaged using a CamScan CS24 scanning electron microscope.

### Immunohistochemistry

Specimens sampled for immunohistochemistry were fixed in 4% paraformaldehyde (PFA) made up in PBS with 9% sucrose for a duration of 1 h at room temperature. The fixation was stopped through rinsing with the same buffer. Subsequently, the specimens were preserved in PBS with 9% sucrose and NaN3. Prior to phalloidin staining, the specimens were preincubated for 1 h in 5% PBT (PBS + 5% Triton X + 0.05% NaN3 + 0.25% bovine serum albumin (BSA) + 7% sucrose), followed by rinsing with 0.5% PBT (PBS + 0.5% Triton X + 0.05% NaN3 + 0.25% BSA + 7% sucrose). The specimens were then subjected to incubation with primary and secondary antibodies (shown in Ref [28].). Alexa Fluor 488 phalloidin (Invitrogen, Carlsbad, USA) was added during the secondary antibody incubation at a concentration of 0.33 μM and allowed to incubate overnight. Following incubation, the samples were rinsed in PBS and subsequently mounted in Vectashield with DAPI (VECTOR LABORATORIES, Burlingame, USA). These prepared object slides were stored at − 20 °C until they were ready for imaging, which was conducted using an OLYMPUS Fluoview FV-1000 confocal laser scanning microscope. The resulting Z-stacks of images were analyzed and documented using Imaris 7.0 and/or ImarisViewer 10.0.1.

### RNA extraction and sequencing

The RNA-Seq experimental setup included five replicates for each of the following developmental stages: early embryos, single fertilized egg cells, 1-day-old larvae, 2-day-old larvae, 3-day-old larvae, 4-day-old larvae, 5-day-old larvae, and 6-day-old larvae (Suppl. Figure 4). Five and six-day-old larvae are considered "competent larvae", indicating their readiness for metamorphosis. These larvae do not exhibit morphological differences [28] or any significant variations in their gene expression profiles (Suppl. Figure 4) and are collectively represented as "competent larvae" in Fig. 3. Furthermore, additional three replicates were collected for the following stages: early adult males (6–8 h post induction of metamorphosis), late adult males (about 4d post induction, the same as 96 + male in Ref [28].), early adult females (1 day post induction), and late adult females (3 weeks old). Chosen larvae follow the developmental staging system described in Ref. [28].

The animals were selected individually, fixed in RNAlater after examination under a microscope, and photographically documented. These samples were then stored at −80 °C. RNA extraction was carried out on single individuals using the ultra-low input SMART-Seq^®^ HT Kit (Takara Bio USA, Inc.), following the manufacturer's provided instructions. Each larva was carefully retrieved from the RNAlater, rinsed with sterile, cold RNAlater, and promptly transferred to the lysis buffer. The samples in the lysis buffer were either immediately processed or stored at −80 °C for up to 5 days. The SMART-Seq HT kit applies a streamlined single-tube protocol for RNA extraction and cDNA synthesis. Prior to library preparation, the concentration of cDNA was estimated using the Qubit^®^ dsDNA HS (High Sensitivity) Assay Kit, and the quality was assessed with an Agilent 2100 Bioanalyzer (Agilent Biosciences) using the high-sensitivity DNA kit. Dual-indexed libraries were prepared using the Nextera^®^ XT DNA library preparation kit (Illumina), with 1 ng of cDNA as input.

The multiplexed libraries were subjected to 150 bp paired-end Illumina sequencing, which was conducted by Genewiz^™^ Agenta Life Sciences (Leipzig, Germany) using a NovaSeq 6000 platform. The average number of reads obtained was 23.2 million, with a range spanning from 1.8 million to 79.3 million reads per sample. Detailed sequencing statistics for each sample can be found in Table S1. To ensure open access to the sequence data, all of the generated data has been deposited in the NCBI Sequence Read Archive (SRA) under the BioProject accession number PRJNA1088276 (Suppl. Table 1).

### Differential gene expression analyses

Paired-end reads were quality assessed with FASTQC v.0.11.8 [44]. For constructing a reference transcriptome assembly, 10 million reads from three replicates of each sequenced stage were subsampled, selecting those with the highest quality. The subsampled reads were merged and subsequently trimmed using TrimmGalore! V.0.6.5 [45]. Parameters were set to analyze paired-end libraries, including the automatic detection of adapter sequences; all other settings were retained at their default values. The reference transcriptome was assembled using Trinity v2.15.1 [46], Computation was performed on the National Life Science Supercomputing Center—Computerome 2.0 (www.computerome.dk). ORFs were identified using TransDecoder v5.7.1 [47] and the transcriptome assembly was annotated following the Trinotate v.4.0.2 annotation suite [48]. Kallisto v0.46.1 [49] was used for read mapping and quantification. The differential gene expression analyses were conducted using the *edgeR* [50] and *limma* [51] R packages. Data were visualized and plotted with the *ggplot2* package in R*.*

### Phylogenetic reconstruction of gene trees

The dataset for metazoan chitin synthases (*CS*s) published in Ref [34]. was downloaded. The two *CS* genes identified within the *Osedax japonicus* transcriptome were aligned with this comprehensive metazoan *CS* dataset. Additionally, *NF70* sequences were extracted from public databases to be analyzed alongside the *NF70* sequence found in the *O. japonicus* transcriptomes. A list of all analyzed sequences is shown in Suppl. Tables 2 and 3. Sequence alignments were executed using MAFFT [52]. Subsequently, maximum likelihood analyses were conducted using IQTree [53] with 1000 ultrafast bootstrap replicates [54]. These analyses were conducted under the best-fit model for each alignment, identified as follows: for CS sequences, [LG + F + I + G4]; and for *NF70* sequences, [LG + I + G4].

### Hybridization chain reaction (HCR)

Larvae intended for the Hybridization Chain Reaction (HCR) procedure were initially fixed in 4% paraformaldehyde (PFA) at room temperature for a duration of 1 h. Following fixation, they were gradually transitioned into 100% methanol and stored at −20 °C until the start of the HCR protocol. The probe wash buffer, hybridization buffer, amplification buffers, and DNA HCR amplifier hairpin sets, were sourced from Molecular Instruments (https://www.molecularinstruments.com). Probes were designed using the custom probe design software, *Insitu Probe Generator* [55]. This software facilitates the creation of custom DNA oligo probe pools that align with established HCR protocols [31, 32], reaction components, and amplification reagents. Specifically, for *Osedax japonicus* Chitin Synthase 1 (*CS1*; Genbank: PP475509) messenger RNA (mRNA), a set of 33 custom probe pairs compatible with B2 amplifier hairpins was designed. For *NF70* (Genbank: PP475511) mRNA, 28 probe pairs compatible with B3 amplifier hairpins were designed. Additionally, for *CS2* (Genbank: PP475510), another set of 31 probe pairs compatible with B1 amplifier hairpins was created. These probes were procured from Integrated DNA Technologies (IDT), and the sequences for the ordered oligo pools can be found in Supplementary Information. The HCR procedure adhered to the published protocol for *Platynereis dumerilii* as outlined by Kuehn et al. in Ref. [55]. During the Proteinase-K digestion step, the digestion time was 60 s at room temperature. At the conclusion of the signal amplification stage (day 3), the samples were rinsed and subsequently mounted on object slides using Vectashield with DAPI (VECTOR LABORATORIES, Burlingame, USA). These prepared object slides were stored at -20 °C until they were ready for imaging, which was carried out using an OLYMPUS Fluoview FV-1000 confocal laser scanning microscope. The resulting Z-stacks of images were analyzed and documented using Imaris 7.0 and/or ImarisViewer 10.0.1.

### Supplementary Information

Supplementary Material 1.

Supplementary Material 2.

Supplementary Material 3.

Supplementary Material 4: Fig 1. Maximum likelihood phylogenetic tree (IQ-TREE) of metazoan and choanoflagellate chitin synthases, with fungal chitin synthases serving as the outgroup. The chitin synthase genes from *Osedax japonicus,* *OjapCS1* and *OjapCS2*, are highlighted in red. These sequences were analyzed in conjunction with the dataset previously published in Zakrzewski et al. [34]. Node support values above 99.0 are omitted for clarity. The labeling and color scheme are consistent with Zakrzewski et al. [34] to facilitate comparison and interpretation.

Supplementary Material 5: Fig 2. Maximum likelihood phylogenetic tree (IQ-TREE) of the neuronal cytoplasmic intermediate filament protein gene *NF70 *in molluscs, annelids and the brachiopod *Lingula anatina*, with the platyhelminth *NF70* group serving as the outgroup. Node support values above 99.0 are omitted for clarity.

Supplementary Material 6: Fig 3. Gene expression patterns of chitin-synthases (*CS1* and *CS2*) and of the neuronal cytoplasmic intermediate filament protein gene *NF70*. Volcano plots showing log fold-change versus adjusted P-values in pairwise comparisons of (**a**) 4-day-old larvae vs. early adult females and (**b**) 4-day-old larvae vs. early adult males.

Supplementary Material 7: Fig 4. Principal component analysis (PCA) of gene expression levels in sampled *Osedax japonicus* stages. PCA was executed with the *prcomp* function in *R*, on log2-transformed normalized data. The first two principal components are plotted. Colors indicate different life stages.

## Data Availability

Sequence data that support the findings of this study have been deposited in GenBank with the accession codes: *CS1*—PP475509; *CS2*—PP475510; *NF70*—PP475511 and the raw RNASeq data are available in SRA-Archive under the BioProject accession number PRJNA1088276.
