# Supplementary material for: Hooked on zombie worms? Genetic blueprints of bristle formation in Osedax japonicus (Annelida)
Source: EvoDevo. 2024 Jun 4;15:7. doi: 10.1186/s13227-024-00227-1 (PMC11149249; doi:10.1186/s13227-024-00227-1)
Supplement: Supplementary file 3 — Supplementary Material 3. [file 13227_2024_227_MOESM3_ESM.doc]

**Suppl. Table 2 -** List of all sequences used in the phylogenetic analysis of chitin synthases. The dataset from Zakrzewski et al. (Ref. ^34^), is expanded with the newly generated sequenced for *Osedax japonicus* are highlighted in bold.

| **Abbreviation**  **in Suppl. Fig. 1** | ***Organism*** | **GenBank or UniProt accession numbers** | **JGI protein IDs or IDs of other genomes** |
| --- | --- | --- | --- |
| *AmquCS1 (XP_003389565)* | *Amphimedon queenslandica* | XP_003389565.1 |  |
| *AmquCS2 (XP_003385441)* | *Amphimedon queenslandica* | XP_003385441.1 |  |
| *AngaCS1 (XP_321336)* | *Anopheles gambiae str.* PEST | XP_321336.5 |  |
| *AngaCS2 (XP_321951)* | *Anopheles gambiae str.* PEST | XP_321951.2 |  |
| *AsfuCSA (B0XTK9)* | *Aspergillus fumigatus* | B0XTK9 |  |
| *AsfuCSB (B0YED9)* | *Aspergillus fumigatus* | B0YED9 |  |
| *AsfuCSC (Q92197)* | *Aspergillus fumigatus* | Q92197 |  |
| *AsfuCSD (P78746)* | *Aspergillus fumigatus* | P78746 |  |
| *AsfuCSE (EDP54849.1)* | *Aspergillus fumigatus* A1163 | EDP54849.1 |  |
| *AsfuCSG (P54267)* | *Aspergillus fumigatus* | P54267 |  |
| *AsorCSA (Q8TGD5)* | *Aspergillus oryzae* | Q8TGD5 |  |
| *AsorCSC (Q8TGD4)* | *Aspergillus oryzae* | Q8TGD4 |  |
| *AsorCSZ (XP_001821795.1)* | *Aspergillus oryzae* | XP_001821795.1 |  |
| *AtriCS (AAY86556)* | *Atrina rigida* | AAY86556.1 |  |
| *BebaHAS (EJP68808.1)* | *Beauveria bassiana ARSEF 2860* | EJP68808.1 |  |
| *BlgrCS (AAF04279.2)* | *Blumeria graminis* | AAF04279.2 |  |
| *Brfl (XP_002586939.1)* | *Branchiostoma floridae* | XP_002586939.1 |  |
| *Brfl1 (XP_002592459)* | *Branchiostoma floridae* | XP_002592459 |  |
| *Brfl2 (XP_002592461)* | *Branchiostoma floridae* | XP_002592461 |  |
| *Brfl3 (XP_002602987)* | *Branchiostoma floridae* | XP_002602987 | 84722 |
| *BrmaCS1 (Q4VW80)* | *Brugia malayi* | Q4VW80 |  |
| *BrmaCS2 (Q9GQC3)* | *Brugia malayi* | Q9GQC3 |  |
| *CaalCS1 (P23316)* | *Candida albicans* | P23316 |  |
| *CaalCS2 (P30572)* | *Candida albicans* | P30572 |  |
| *CateCS1 (51996)* | *Capitella teleta* | gw1.226.38.1 | 51996 |
| *CateCS2 (22434)* | *Capitella teleta* | gw1.448.3.1 | 22434 |
| *CateCS3 (104090)* | *Capitella teleta* | e_gw1.662.3.1 | 104090 |
| *CateCS4 (126651)* | *Capitella teleta* | e_gw1.691.2.1 | 126651 |
| *CeelCS1 (AAX62732)* | *Caenorhabditis elegans* | AAX62732 |  |
| *CeelCS2 (AAX62733)* | *Caenorhabditis elegans* | AAX62733 |  |
| *Ciin (291898)* | *Ciona intestinalis* |  | 291898 |
| *CogrCS (310792055)* | *Colletotrichum graminicola* | 310792055 |  |
| *CogrCS (EFQ28255.1)* | *Colletotrichum graminicola* | EFQ28255.1 |  |
| *CogrCS (EFQ29368.1)* | *Colletotrichum graminicola* | EFQ29368.1 |  |
| *CogrCSA (AAL23717.1)* | *Colletotrichum graminicola* | AAL23717.1 |  |
| *CogrCSA (AAL55424.1)* | *Colletotrichum graminicola* | AAL55424.1 |  |
| *CogrCSC (AAL23719.1)* | *Colletotrichum graminicola* | AAL23719.1 |  |
| *CopoCS7 (AAQ10290.1)* | *Coccidioides posadasii* | AAQ10290.1 |  |
| *Dare1 (XP_691803)* | *Danio rerio* | XP_691803.3 |  |
| *Dare2 (XP_691594)* | *Danio rerio* | XP_691594.3 |  |
| *Dare3 (XP_001921014)* | *Danio rerio* | XP_001921014.3 |  |
| *DareHAS (NP_775327)* | *Danio rerio* | NP_775327.1 |  |
| *DiimCS (Q9GQ90)* | *Dirofilaria immitis* | Q9GQ90 |  |
| *DrmeCS1 (NP_524233)* | *Drosophila melanogaster* | NP_524233 |  |
| *DrmeCS2 (NP_001137997)* | *Drosophila melanogaster* | NP_001137997 |  |
| *FuoxHAS (ENH63163.1)* | *Fusarium oxysporum f. sp. cubense race 1* | ENH63163.1 |  |
| *GagaHAS (AAF14347)* | *Gallus gallus* | AAF14347.1 |  |
| *HymaCS (XP_002162504)* | *Hydra magnipapillata* | XP_002162504.1 |  |
| *LeasCS1* | *Leptochiton asellus* | KJ405451.1 |  |
| *LeasCS2* | *Leptochiton asellus* | KJ405452.1 |  |
| *LecoCSa* | *Leucosolenia complicata* | KJ405453.1 |  |
| *LecoCSb* | *Leucosolenia complicata* | KJ405454.1 |  |
| *LeedCS1 (A0PAB0)* | *Lentinula edodes* | A0PAB0 |  |
| *LogiCS10 (208748)* | *Lottia gigantea* |  | 208748 |
| *LogiCS2 (127716)* | *Lottia gigantea* |  | 127716 |
| *LogiCS3 (235019)* | *Lottia gigantea* |  | 235019 |
| *LogiCS4 (127517)* | *Lottia gigantea* |  | 127517 |
| *LogiCS5 (154879)* | *Lottia gigantea* |  | 154879 |
| *LogiCS6 (111741)* | *Lottia gigantea* |  | 111741 |
| *LogiCS7 (91560)* | *Lottia gigantea* |  | 91560 |
| *LogiCS8 (91642)* | *Lottia gigantea* |  | 91642 |
| *LogiCS9 (143023)* | *Lottia gigantea* |  | 143023 |
| *MacrCS* | *Macandrevia cranium* | KJ405469.1 |  |
| *MagrCS7 (ACH58563.1)* | *Magnaporthe grisea* | ACH58563.1 |  |
| *MaseCS1 (EC 2.4.1.16)* | *Manduca sexta* | EC 2.4.1.16 |  |
| *MaseCS2 (AAX20091)* | *Manduca sexta* | AAX20091.1 |  |
| *MearCS (Q8T5G8)* | *Meloidogyne artiellia* | Q8T5G8 |  |
| *MobrCS (XP_001743227)* | *Monosiga brevicollis* MX1 | XP_001743227.1 |  |
| *MyciCS* | *Myzostoma cirriferum* | KJ405468.1 |  |
| *MygaCS (ABQ08059)* | *Mytilus galloprovincialis* | ABQ08059.1 |  |
| *NecrCS1 (P29070)* | *Neurospora crassa* | P29070 |  |
| *NecrCS2 (P30589)* | *Neurospora crassa* | P30589 |  |
| *NecrCS3 (P30588)* | *Neurospora crassa* | P30588 |  |
| *NecrCS4 (8117)* | *Neurospora crassa* |  | 8117 |
| *NecrCSD (XP_961758.2)* | *Neurospora crassa* | XP_961758.2 |  |
| *NeveCS1 (XP_001633545)* | *Nematostella vectensis* | XP_001633545.1 |  |
| *NeveCS2 (XP_001637059)* | *Nematostella vectensis* | XP_001637059.1 |  |
| ***Ojap CS1*** | ***Osedax japonicus*** | **PP475509** |  |
| ***Ojap CS2*** | ***Osedax japonicus*** | **PP475510** |  |
| *OsfuCS1 (ACB13821)* | *Ostrinia furnacalis* | ACB13821.1 |  |
| *OsfuCS2 (ABX46067)* | *Ostrinia furnacalis* | ABX46067.1 |  |
| *OwfuCS1* | *Owenia fusiformis* | KJ405455.1 |  |
| *OwfuCS2* | *Owenia fusiformis* | KJ405456.1 |  |
| *OwfuCS3* | *Owenia fusiformis* | KJ405457.1 |  |
| *OwfuCS4* | *Owenia fusiformis* | KJ405458.1 |  |
| *OwfuCS5* | *Owenia fusiformis* | KJ405459.1 |  |
| *OwfuCS6* | *Owenia fusiformis* | KJ405467.1 |  |
| *PabrCS2 (EEH43093.1)* | *Paracoccidioides brasiliensis* Pb17 | EEH43093.1 |  |
| *PabrCS4 (ABV31248.1)* | *Paracoccidioides brasiliensis* Pb17 | ABV31248.1 |  |
| *PifuCS (BAF73720)* | *Pinctada fucata* | BAF73720.1 |  |
| *PlduCS1* | *Platynereis dumerilii* | KJ405460.1 |  |
| *PlduCS2* | *Platynereis dumerilii* | KJ405461.1 |  |
| *PlduCS3* | *Platynereis dumerilii* | KJ405470.1 |  |
| *PugrCS (E3JVL1)* | *Puccinia graminis f. sp. tritici* | E3JVL1 |  |
| *PugrCS (E3JZ94)* | *Puccinia graminis f. sp. tritici* | E3JZ94 |  |
| *PugrCS (E3L5J0)* | *Puccinia graminis f. sp. tritici* | E3L5J0 |  |
| *PugrCS4 (E3KJ13)* | *Puccinia graminis f. sp. tritici* | E3KJ13 |  |
| *SaalCS1* | *Sabellaria alveolata* | KJ405462.1 |  |
| *SaalCS2* | *Sabellaria alveolata* | KJ405471.1 |  |
| *SaalCS3* | *Sabellaria alveolata* | KJ405463.1 |  |
| *SaceCS1 (P08004)* | *Saccharomyces cerevisiae* | P08004 |  |
| *SaceCS2 (P14180)* | *Saccharomyces cerevisiae* | P14180 |  |
| *SaceCS3 (P29465.3)* | *Saccharomyces cerevisiae* | P29465.3 |  |
| *SaspCS (EGD80959)* | *Salpingoeca sp.* ATCC 50818 | EGD80959.1 |  |
| *SyciCS* | *Sycon ciliatum* | KJ405464.1 |  |
| *ThpsCS1 (6575)* | *Thalassiosira pseudonana* |  | 6575 |
| *ThpsCS2 (7305)* | *Thalassiosira pseudonana* |  | 7305 |
| *ThpsCS3 (4368)* | *Thalassiosira pseudonana* |  | 4368 |
| *ThpsCS4 (4413)* | *Thalassiosira pseudonana* |  | 4413 |
| *ThroCS (ACL00587.1)* | *Thalassiosira rotula* | ACL00587.1 |  |
| *TrcaCS1 (NP_001034491)* | *Tribolium castaneum* | NP_001034491.1 |  |
| *TrcaCS2 (NP_001034492)* | *Tribolium castaneum* | NP_001034492.1 |  |
| *TrspCS (XP_003378777)* | *Trichinella spiralis* | XP_003378777.1 |  |
| *UsmaCS1 (P30598)* | *Ustilago maydis* | P30598 |  |
| *UsmaCS2 (P30599)* | *Ustilago maydis* | P30599 |  |
| *UsmaCS4 (Q99127)* | *Ustilago maydis* | Q99127 |  |
| *Xetr (XP_002942397)* | *Xenopus tropicalis* | XP_002942397.1 |  |

**Suppl. Table 3 -** List of all sequences used in the phylogenetic analysis of NF70. Newly generated sequenced for *Osedax japonicus* are highlighted in bold.

| ***Organism*** | **GenBank or UniProt accession numbers** | **JGI protein IDs or IDs of other genomes** |
| --- | --- | --- |
| *Batillaria attramentaria* | KAG5693148 | BaRGS_035346 |
| *Crassostrea gigas* | XP_034300507 |  |
| *Dimorphilus gyrociliatus* | CAD5124847.1 | DgyrCDS13102 |
| *Doryteuthis pealeii* |  | NF70_DORPE |
| *Dreissena polymorpha* | XP_052221383 |  |
| *Echinococcus granulosus* | KAH9286247 |  |
| *Fasciola hepatica* | THD20852 |  |
| *Haliotis rufescens* | XP_046340225 |  |
| *Helobdella robusta* | XP_009030018.1 |  |
| *Hirudo medicinalis* | AAD29248.1 |  |
| *Lingula anatina* | XP_013412658 |  |
| *Lumbricus terrestris* | CAA58705.1 |  |
| *Mercenaria mercenaria* | XP_045195495 |  |
| *Mercenaria mercenaria* | XP_053380999 |  |
| *Mizuhopecten yessoensis* | OWF48901 |  |
| *Mya arenaria* | XP_052789036 |  |
| *Mya arenaria* | XP_052790611 |  |
| *Octopus bimaculoides* | XP_052831305 |  |
| *Octopus sinensis* | XP_029648899 |  |
| *Octopus vulgaris* | CAI9738634 |  |
| ***Osedax japonicus*** | **PP475511** |  |
| *Owenia fusiformis* | CAH1785440.1 |  |
| *Phascolion stombus* | CAB38180.1 |  |
| *Physella acuta* | XP_059165213 |  |
| *Ridgeia piscesae* | KAK2175754.1 |  |
| *Schistosoma japonicum* | TNN07284 |  |
| *Ylistrum balloti* | XP_060081254 |  |
