# Supplementary figures and images for: Hooked on zombie worms? Genetic blueprints of bristle formation in Osedax japonicus (Annelida)
Source: EvoDevo. 2024 Jun 4;15:7. doi: 10.1186/s13227-024-00227-1 (PMC11149249; doi:10.1186/s13227-024-00227-1)

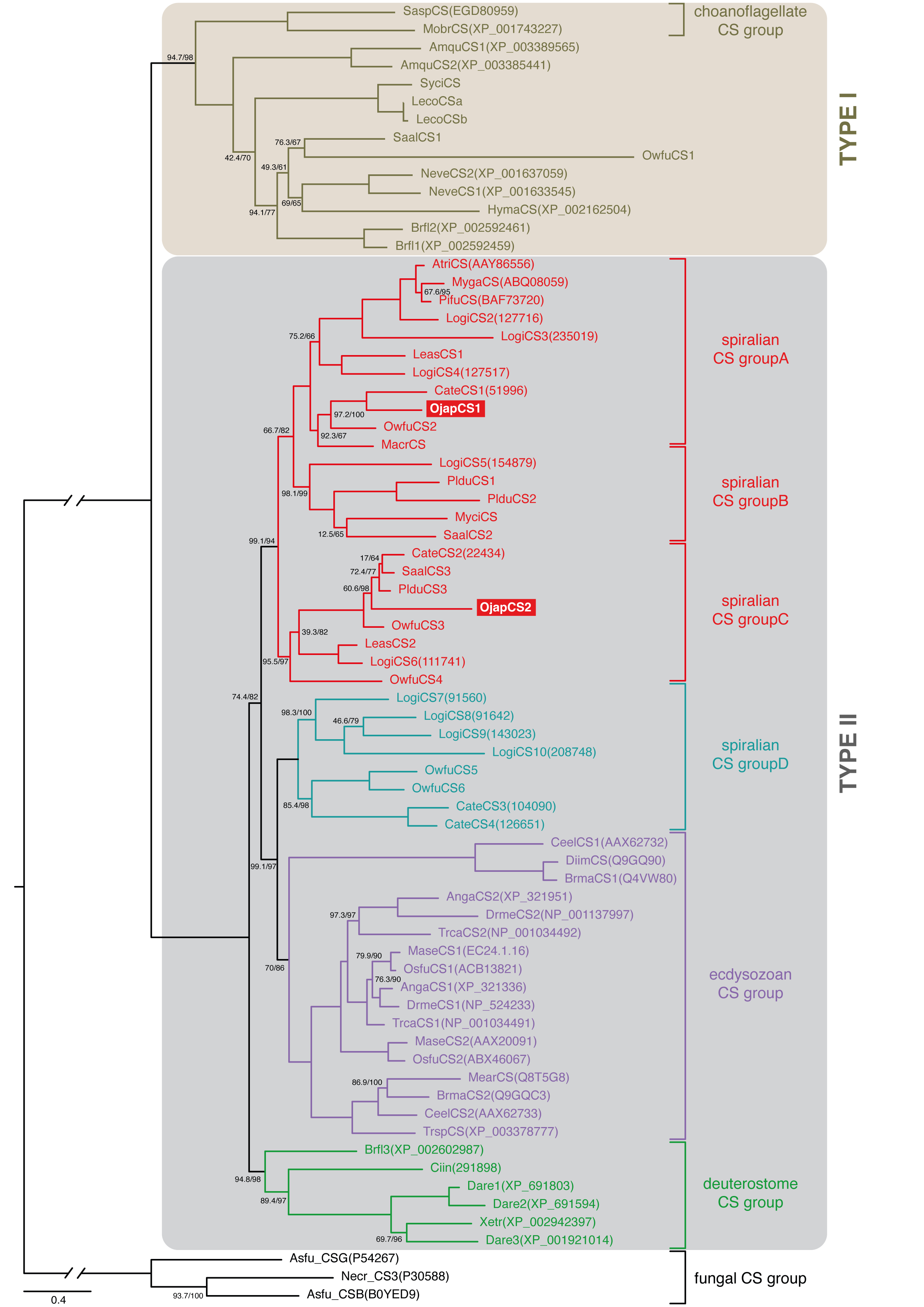

Supplement: Supplementary file 4 — Supplementary Material 4: Fig 1. Maximum likelihood phylogenetic tree (IQ-TREE) of metazoan and choanoflagellate chitin synthases, with fungal chitin synthases serving as the outgroup. The chitin synthase genes from Osedax japonicus, OjapCS1 and OjapCS2, are highlighted in red. These sequences were analyzed in conjunction with the dataset previously published in Zakrzewski et al. [34]. Node support values above 99.0 are omitted for clarity. The labeling and color scheme are consistent with Zakrzewski et al. [34] to facilitate comparison and interpretation. [file 13227_2024_227_MOESM4_ESM.tif]

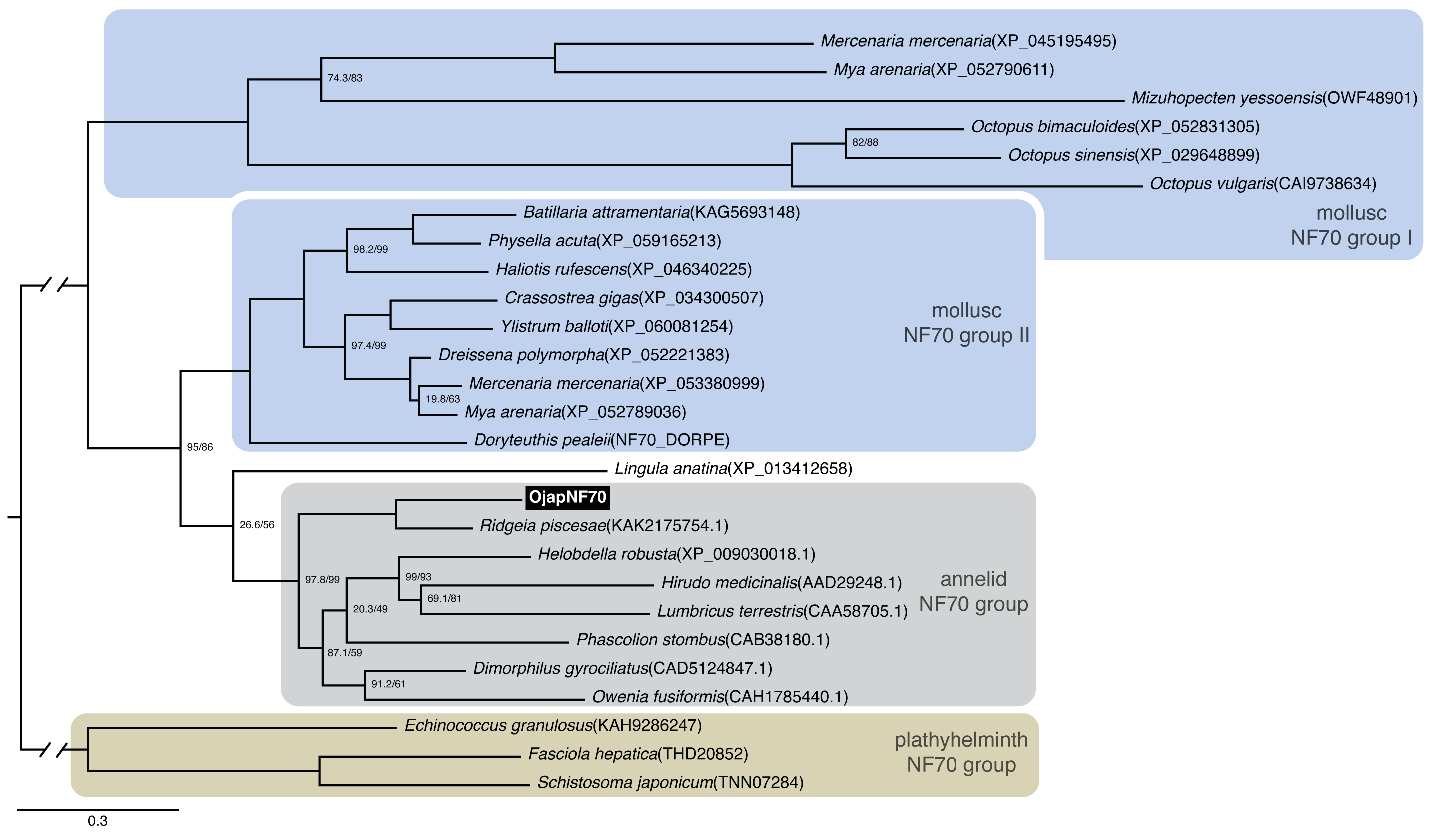

Supplement: Supplementary file 5 — Supplementary Material 5: Fig 2. Maximum likelihood phylogenetic tree (IQ-TREE) of the neuronal cytoplasmic intermediate filament protein gene NF70 in molluscs, annelids and the brachiopod Lingula anatina, with the platyhelminth NF70 group serving as the outgroup. Node support values above 99.0 are omitted for clarity. [file 13227_2024_227_MOESM5_ESM.tif]

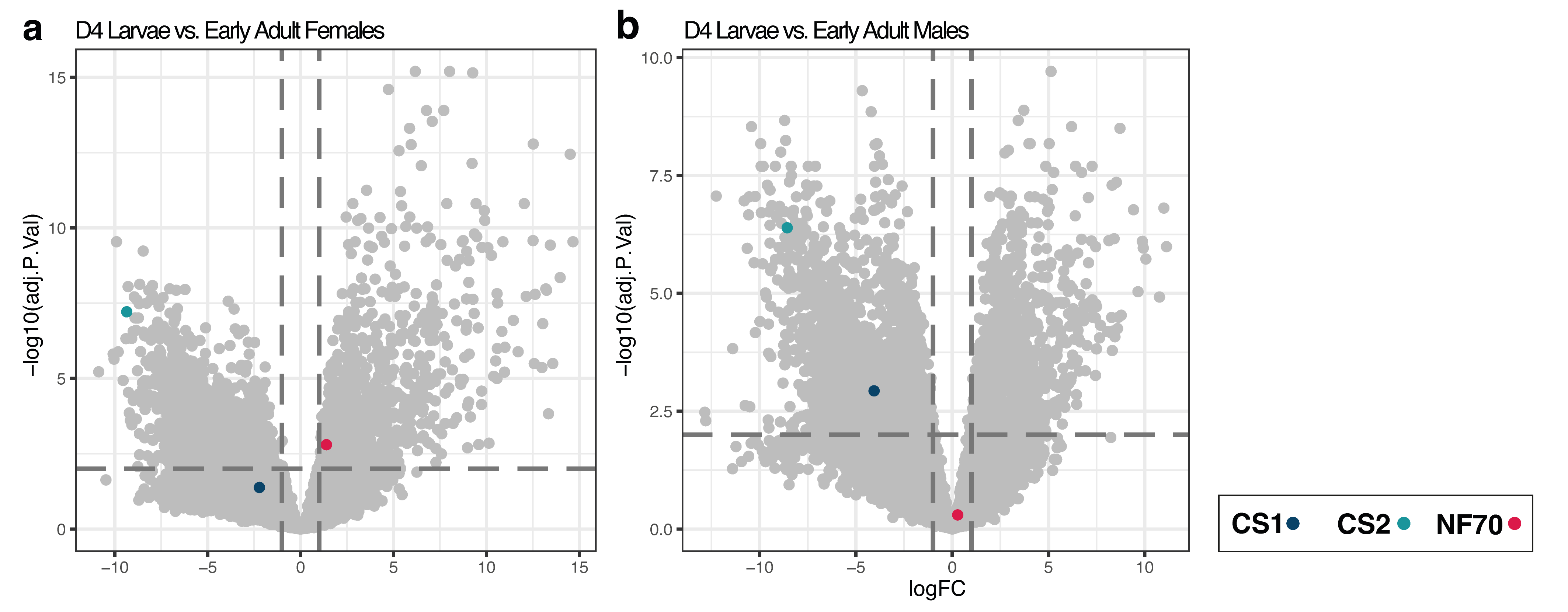

Supplement: Supplementary file 6 — Supplementary Material 6: Fig 3. Gene expression patterns of chitin-synthases (CS1 and CS2) and of the neuronal cytoplasmic intermediate filament protein gene NF70. Volcano plots showing log fold-change versus adjusted P-values in pairwise comparisons of (a) 4-day-old larvae vs. early adult females and (b) 4-day-old larvae vs. early adult males. [file 13227_2024_227_MOESM6_ESM.tif]

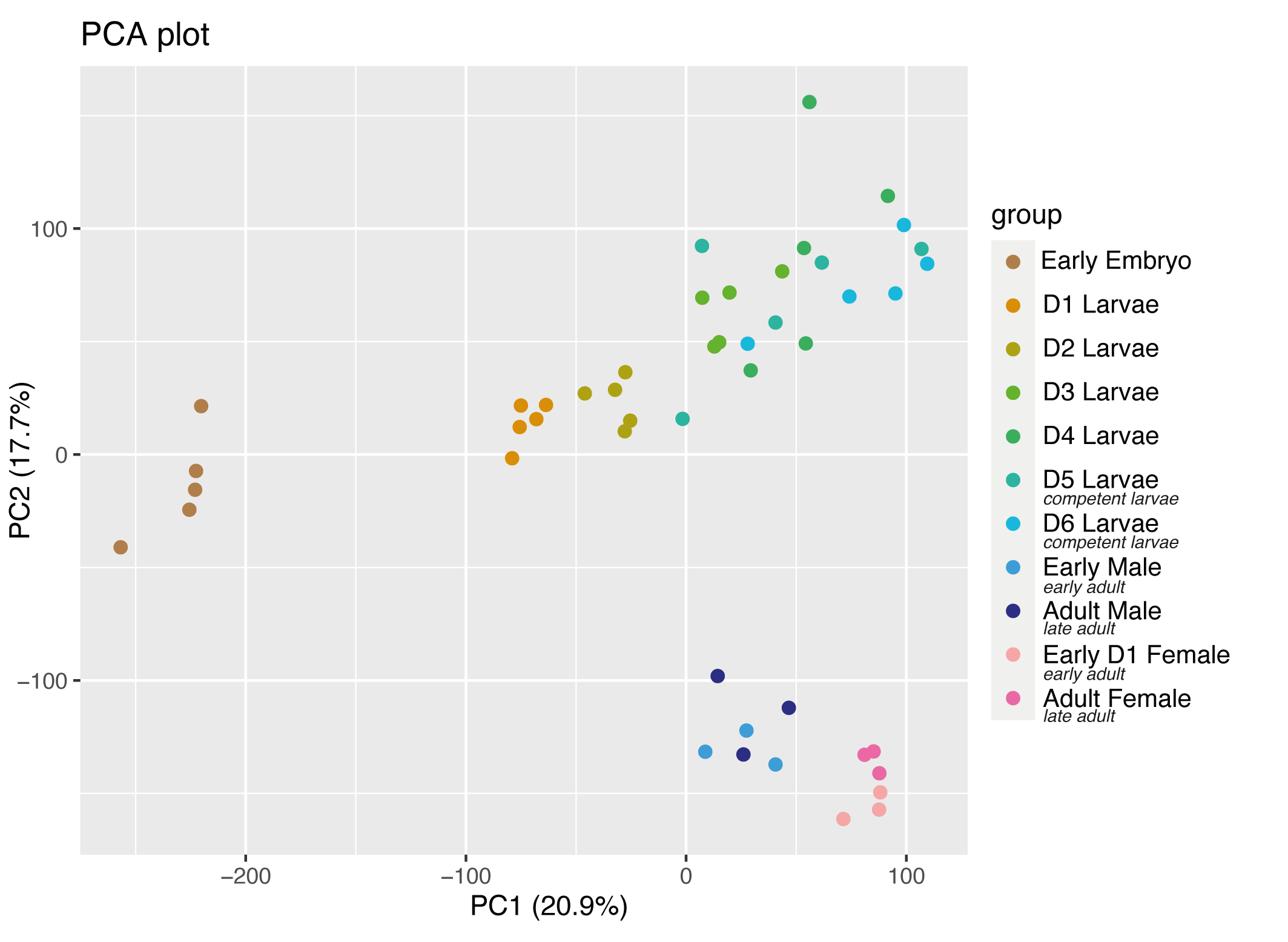

Supplement: Supplementary file 7 — Supplementary Material 7: Fig 4. Principal component analysis (PCA) of gene expression levels in sampled Osedax japonicus stages. PCA was executed with the prcomp function in R, on log2-transformed normalized data. The first two principal components are plotted. Colors indicate different life stages. [file 13227_2024_227_MOESM7_ESM.tif]
